# Food-Derived Zinc-Chelating Peptides: Coordination Chemistry, Intestinal Transport, and Nutritional Functionality

**DOI:** 10.3390/nu18152409

**Published:** 2026-07-23

**Authors:** Lanshi Tian, Shan Yang, Peng Li, Jinzi Yin, Jia Zhou, Zhongmei He

**Affiliations:** College of Traditional Chinese Medicines, Jilin Agricultural University, No. 2888, Xincheng Street, Changchun 130118, China; 18205037129@163.com (L.T.); 15761404093@163.com (S.Y.); 18337422670@163.com (P.L.); 18040289345@163.com (J.Y.)

**Keywords:** food-derived peptides, zinc-chelating peptides, intestinal transport, zinc homeostasis, precision nutrition

## Abstract

Food-derived zinc-chelating peptides (ZCPs) have emerged as promising nutritional carriers that enhance zinc bioavailability and exert diverse biological activities. Compared with conventional zinc supplements, peptide-mediated zinc delivery systems exhibit superior gastrointestinal stability, reduced mineral precipitation, and improved intestinal transport efficiency. The present study demonstrates that ZCPs regulate zinc homeostasis through multiple mechanisms, including coordination chemistry, transporter-mediated absorption, regulation of the epithelial barrier, and interactions with the intestinal microenvironment. Beyond facilitating zinc absorption, peptide–zinc complexes exhibit antioxidant, anti-inflammatory, immunomodulatory, metabolic regulatory, and gut-protective activities through the synergistic effects of coordinated zinc ions and peptide bioactivity. Advances in spectroscopic characterization, computational modeling, and systems nutrition approaches have further expanded current understanding of peptide–zinc coordination behavior and physiological functionality. Nevertheless, current studies remain limited by insufficient clinical validation and an incomplete understanding of intestinal transport kinetics and controlled zinc release mechanisms. Overall, food-derived ZCPs demonstrate considerable potential for precision nutrition interventions and the development of next-generation functional zinc delivery systems.

## 1. Introduction

Zinc is an essential trace element involved in numerous biological processes, including immune regulation, antioxidant defense, intestinal barrier maintenance, cellular signaling, and metabolic homeostasis [1]. Insufficient zinc intake or impaired zinc absorption is closely associated with immune dysfunction, oxidative stress, intestinal disorders, metabolic imbalance, and growth impairment [2]. Despite the widespread use of zinc supplementation strategies, the bioavailability of conventional zinc salts remains limited due to poor gastrointestinal stability, precipitation with dietary inhibitors, competitive mineral interactions, and gastrointestinal irritation.

To overcome these limitations, increasing attention has been directed toward food-derived zinc-chelating peptides (ZCPs) as emerging zinc delivery systems [3]. These peptides are typically generated through enzymatic hydrolysis of dietary proteins and contain functional amino acid residues capable of coordinating zinc ions into relatively stable complexes. Compared with free zinc ions, peptide-mediated zinc delivery may improve zinc solubility, gastrointestinal stability, and epithelial transport efficiency while reducing unwanted precipitation within the intestinal lumen [4].

Recent studies further suggest that the nutritional behavior of peptide–zinc complexes is governed not only by zinc-binding affinity but also by gastrointestinal digestion dynamics, transporter accessibility, controlled zinc release, and interactions with the gut microenvironment [5]. Peptide–zinc systems may participate in multiple biological processes involving oxidative stress regulation, intestinal barrier protection, immune homeostasis, and metabolic modulation [6]. However, despite rapid advances in peptide screening and structural characterization, several important mechanistic questions remain unresolved. In particular, whether intact peptide–zinc complexes can survive gastrointestinal digestion and undergo direct transporter-mediated absorption remains controversial. In addition, most current evidence still relies heavily on simplified in vitro models and animal studies, while clinically relevant bioavailability evaluation remains insufficient [7].

Therefore, this review summarizes recent advances in the dietary sources, coordination mechanisms, intestinal transport pathways, and nutritional functions of food-derived ZCPs. Current limitations and future perspectives involving computational prediction, multi-omics analysis, and advanced delivery systems are also discussed to provide insights into the development of next-generation zinc nutrition strategies.

## 2. Methods

**Figure 1:**
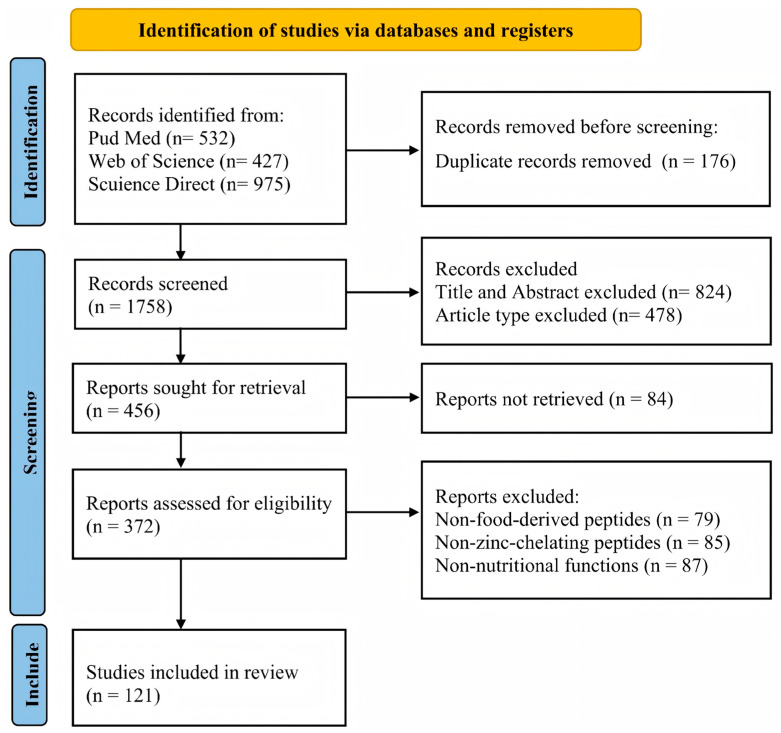
PRISMA flowchart of the literature screening process.

A comprehensive literature review was conducted to investigate the therapeutic and nutritional potential of food-derived ZCPs as emerging zinc supplementation systems in nutrition and disease-related research. The databases utilized included PubMed, Web of Science, and ScienceDirect (up to March 2026). Relevant studies were identified using the keywords food-derived peptides, ZCPs, peptide–zinc complexes, zinc bioavailability, intestinal transport, ZIP transporters, PepT1, and nutritional functionality. This review summarizes the dietary sources of food-derived ZCPs and discusses their zinc-binding mechanisms, intestinal absorption pathways, and multifunctional biological activities, with particular emphasis on zinc homeostasis regulation, antioxidant activity, intestinal barrier modulation, immunoregulation, and metabolic regulation. Our aim was to provide researchers with a comprehensive overview of recent advances in peptide-mediated zinc delivery systems and to offer insights into zinc nutritional supplementation and potential therapeutic applications based on current findings. This review was prepared according to the PRISMA guidelines [8], and the literature screening process is presented in Figure 1. The screening procedure followed predefined exclusion criteria to minimize potential bias. Specifically, (1) non-food-derived peptides referred to peptide systems lacking dietary protein origins; (2) non-zinc-chelating peptides referred to studies without confirmed zinc-binding activity; and (3) non-nutritional functions referred to studies unrelated to zinc bioavailability or nutritional regulation. These studies were excluded during the full-text screening stage to ensure the reliability and nutritional relevance of the included evidence.

## 3. Dietary Sources of Zinc-Chelated Peptides

**Figure 2:**
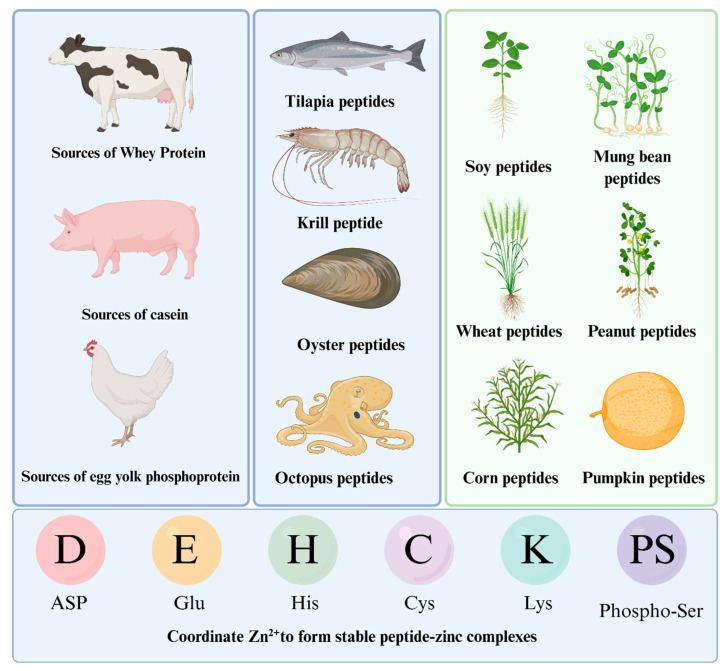
Dietary peptide sources and key zinc-binding amino acid residues involved in peptide–zinc complex formation.

ZCPs are widely derived from animal, aquatic, and plant proteins through enzymatic hydrolysis [9]. These peptides generally contain zinc-binding amino acid residues, including glutamic acid (Glu), aspartic acid (ASP), histidine (His), cysteine (Cys), lysine (Lys), and phosphorylated serine (pSer), which enable the formation of stable peptide–zinc complexes with enhanced solubility and bioavailability [10]. Compared with conventional zinc salts, food-derived peptides not only improve zinc stability and intestinal transport but may also provide additional antioxidant, anti-inflammatory, and metabolic regulatory activities (Figure 2).

### 3.1. Terrestrial Animal Sources

Animal-derived proteins are among the most extensively studied sources of ZCPs due to their balanced amino acid composition and high digestibility. Milk proteins, particularly casein and whey protein, have demonstrated strong zinc-binding capacity. Casein phosphopeptides (CPPs), generated through enzymatic hydrolysis, contain abundant phosphorylated serine residues that provide multiple negatively charged coordination sites for zinc binding, thereby improving zinc solubility and resistance to precipitation under gastrointestinal conditions [11]. Several zinc-chelating peptide sequences identified from casein hydrolysates, namely two peptide fragments Tyr-Pro-Val-Glu-Pro-Phe and Glu-Met-Pro-Phe-Pro-Lys, exhibit high zinc-binding capacity and enhanced intestinal transport [12].

Egg yolk phosphoprotein-derived phosphopeptides also possess strong zinc-chelating ability due to their dense phosphoserine clusters. These phosphate groups coordinate zinc ions through multidentate interactions, generating stable peptide–zinc complexes with favorable gastrointestinal stability and bioavailability [13]. In addition, whey protein-derived peptides can chelate zinc through amino, carboxyl, and amide groups, forming stable coordination structures that improve zinc stability and absorption efficiency [14].

### 3.2. Aquatic Animal Sources

Aquatic proteins and processing byproducts represent important sources of ZCPs because of their high protein content and abundant metal-binding amino acids. Marine-derived peptides have attracted increasing attention due to their combined mineral-binding and bioactive properties. Tilapia skin collagen hydrolysates have yielded several ZCPs, including Gly-Phe-Leu-Gly-Ser-Pro, Phe-His-Gly-Leu-Arg, and Leu-Gly-Pro-Tyr, among which Gly-Phe-Leu-Gly-Ser-Pro exhibited strong zinc-binding activity [15]. Oyster-derived peptides also demonstrate excellent zinc-chelating potential, particularly sequences enriched in acidic amino acids such as Asp and Glu, which contribute to improved zinc solubility and gastrointestinal stability [16]. Similarly, sea cucumber-derived peptides containing residues such as Glu, Asp, and His have shown favorable zinc-chelating capacity and structural stability [17]. Antarctic krill peptides have additionally been reported to form peptide–zinc complexes with greater digestive stability than conventional zinc salts under simulated gastrointestinal conditions [18].

### 3.3. Plant Sources

Plant-derived ZCPs are increasingly recognized as sustainable and cost-effective zinc carriers suitable for functional foods and plant-based nutrition. Soy protein is one of the most widely studied plant sources due to its balanced amino acid composition and high abundance of acidic amino acid residues involved in zinc coordination. Several ZCPs identified from soybean hydrolysates, including Lys-Tyr-Lys-Arg-Gln-Arg-Trp, Glu-Glu-Gly-Gln-Gln-Gln-Gly-Glu-Gln-Arg, and Val-Phe-Asp-Gly-Glu-Leu-Gln-Glu-Gly-Arg, exhibit strong zinc-binding activity and favorable transport properties [19].

Mung bean proteins also represent promising sources of multifunctional ZCPs. Hydrolysates generated from mung bean protein contain peptides with antioxidant activity, mineral-binding capacity, and potential metabolic regulatory effects. The peptide Ser-Ser-Glu-Asp-Gln-Pro-Phe-Asn-Leu-Arg has been identified as a representative zinc-binding sequence with excellent chelating ability [20].

Overall, current ZCPs are primarily derived from food proteins and underutilized protein-rich byproducts containing favorable zinc-binding amino acid residues or functional peptide sequences (Table 1). Recent advances in computational prediction, virtual proteolysis, and AI-assisted peptide screening may further accelerate the discovery of novel peptide sequences with optimized zinc-chelating and nutritional properties.

## 4. Coordination Mechanisms and Structural Characterization

Accurate characterization of peptide–zinc interactions is essential for understanding coordination mechanisms and predicting biological behavior. In recent years, multiple spectroscopic, computational, and thermodynamic techniques have been employed to elucidate peptide–zinc coordination structures at the molecular level.

### 4.1. Carboxyl–Zinc Chelation Mechanism

Carboxyl-mediated peptide–zinc chelation is the primary and most common mechanism by which food-derived bioactive peptides form coordinate bonds with zinc ions. The β-carboxyl group of Asp, Glu, and the free carboxyl group at the C-terminus of the polypeptide chain undergo deprotonation and activation under near-neutral physiological conditions, transforming neutral COOH groups into negatively charged carboxylate ions, endowing the oxygen atoms with sufficient lone pair electrons to exert strong electrostatic attraction [6]. Under the influence of an electric field, the charged Zn^2+^ ion moves directionally toward the carboxyl-rich region. Upon entering the coordination distance, the oxygen atom on the carboxylate ion precisely injects its lone pair of electrons into the vacant valence orbitals of Zn^2+^, forming a highly directional and strongly bonded O → Zn covalent coordination bond. In this process, a single carboxyl group can form a monodentate coordination with Zn^2+^ via a single oxygen atom, while two adjacent or spatially close carboxyl groups can jointly bind Zn^2+^ via two oxygen atoms, forming stable four- or five-membered chelate ring structures. This chelation effect significantly enhances the thermodynamic stability of the complex [34]. When multiple acidic amino acid residues are distributed consecutively or intermittently along the peptide chain, multiple carboxyl groups can simultaneously participate in coordination, forming multidentate chelate structures that further enhance resistance to acid hydrolysis, enzymatic hydrolysis, and precipitation; The resulting ZCPs is electrically neutral or weakly anionic, making it less prone to precipitation by phytic acid, oxalic acid, phosphate ions, and other substances in the gastrointestinal tract. It can be efficiently absorbed in its intact chelated form via intestinal peptide transporters, thereby significantly improving zinc’s solubility, stability, and bioavailability [35]. Structural analysis of two novel peptides, Val-Asp-Leu-Val-Val-Pro-Arg-Gly-Leu (1164.45 Da) and Leu-Asp-Arg-Leu-Glu (644.77 Da), revealed that zinc ions primarily bind to their amino and carboxyl groups [26]. Characterization of zinc-chelated nanoparticle composites formed from oyster hydrolysates revealed that carboxyl groups are also the primary chelation sites [36].

### 4.2. Phosphate–Zinc Chelation Model

The phosphate groups carried by phosphorylated serine residues on the polypeptide chain form phosphate ions with a high density of negative charge. The multiple oxygen atoms in these groups possess abundant lone pairs of electrons and strong coordinating activity, which concentrate Zn^2+^ near the phosphate groups and form strong covalent coordination bonds [37,38]. A single phosphate group can bind to Zn^2+^ via monodentate or bidentate coordination. However, when a continuous phosphorylated serine sequence is present in the polypeptide chain, multiple phosphate groups can participate in coordination simultaneously, forming multidentate coordination and stable chelate ring structures, which is similar to the carboxyl–zinc coordination mode. This chelation mechanism effectively masks the precipitation effects of antinutritional factors such as phytic acid, oxalic acid, and free phosphate ions on Zn^2+^, enabling the zinc–phosphopeptide chelate to maintain high solubility and structural integrity throughout the gastrointestinal digestive process. It can also be efficiently absorbed via specific intestinal transport pathways, ultimately significantly improving the bioavailability and tissue deposition efficiency of Zn^2+^ [39]. For example, a study reported that under physiologically relevant conditions, CPPs retain zinc in solution in a dose-dependent manner; no low-molecular-weight zinc or calcium chelates derived from the addition of CPPs were detected in 3 kDa ultrafiltration. Furthermore, casein phosphopeptide–zinc chelates can deliver bioavailable nanoparticle zinc in an in vitro mouse jejunal loop model and in polarized Caco-2 cells [40].

### 4.3. Amino–Zinc Chelation Mode

The free amino group at the *N*-terminus of a polypeptide chain and, less frequently, the ε-amino group of lysine residues can participate in Zn^2+^ coordination under near-neutral to weakly basic conditions [41]. A single amino group typically binds to Zn^2+^ in a monodentate manner; however, due to the weak coordinating ability of the amino group itself, it often acts synergistically with nearby carboxyl groups, peptide bond carbonyl oxygen, imidazole groups, and other sites in natural peptides to form bidentate or polydentate mixed coordination structures, generating stable five- or six-membered chelate rings [42]. Three novel non-toxic peptides—Thr-Gly-Leu-Leu, Tyr-Ile-Leu-Ile-Lys, and Phe-Asp-Glu-Glu. ZCPs were prepared under conditions of pH 7.6 and 27.0 °C for 50 min. Structural analysis indicated that both the amino and carboxyl groups of the tetrapeptide Phe-Asp-Glu-Glu serve as major binding sites for zinc ions [30]. Characterization of a novel hydrolyzed chestnut protein zinc complex revealed that zinc binding occurs through coordination with the carboxyl group, the nitrogen atom of the amide bond, and the nitrogen atom of the amino group, inducing conformational changes in the protein hydrolysate [43].

### 4.4. Spectroscopic Characterization

Fourier-transform infrared spectroscopy (FTIR) is one of the most widely used techniques for identifying zinc-binding functional groups. Shifts in absorption peaks corresponding to carboxyl, amino, phosphate, or amide groups are commonly interpreted as evidence of coordination [44]. Similarly, X-ray photoelectron spectroscopy (XPS) can provide information regarding elemental valence states and coordination environments by analyzing binding energy changes following zinc complexation [45]. Additional techniques such as ultraviolet–visible spectroscopy, fluorescence spectroscopy, Raman spectroscopy, circular dichroism, and nuclear magnetic resonance have also been employed to investigate conformational changes and interaction mechanisms [46]. These methods collectively reveal that zinc coordination often induces alterations in peptide secondary structure, hydrophobic exposure, and molecular folding [47]. However, many current studies rely excessively on indirect spectroscopic evidence, particularly FTIR peak shifts, without comprehensive validation using complementary analytical approaches. As a result, proposed coordination models are sometimes speculative or inconsistent across studies.

### 4.5. Thermodynamic and Structural Analysis

Isothermal titration calorimetry has increasingly been used to quantify zinc-binding thermodynamics, including binding constants, enthalpy changes, and stoichiometric ratios. Such analyses provide important insights into coordination strength and driving forces underlying peptide–zinc interactions [48,49]. Meanwhile, electron microscopy and particle size analysis can evaluate nanoparticle formation, aggregation behavior, and structural morphology following zinc chelation [50]. Some peptide–zinc complexes self-assemble into nanoscale structures that may influence gastrointestinal transport and biological activity.

### 4.6. Computational Approaches

Computational modeling has emerged as a powerful tool for studying peptide–zinc interactions. Molecular docking, density functional theory, and molecular dynamics simulations are increasingly used to predict coordination sites, binding energies, conformational stability, and zinc release behavior [51]. These approaches enable visualization of peptide–zinc interactions at atomic resolution and facilitate the rational design of high-affinity zinc-binding peptides [52]. Nevertheless, current computational studies remain limited by simplified simulation environments and insufficient experimental validation. Many models fail to account for gastrointestinal digestion, competitive ion interactions, mucus barriers, and dynamic intestinal transport processes.

### 4.7. Representative Three-Dimensional Coordination Conformations of Peptide–Zinc Complexes

The zinc-chelating behavior of food-derived peptides is strongly influenced by the spatial arrangement of zinc-binding residues and the resulting three-dimensional conformations of peptide–zinc complexes. Although high-resolution crystal structures of peptide–zinc complexes remain limited, spectroscopic characterization combined with molecular docking, density functional theory (DFT), and molecular dynamics (MD) simulations has provided important insights into their coordination geometries and conformational features. Current evidence indicates that Zn^2+^ commonly coordinates with oxygen and nitrogen donor atoms originating from carboxyl groups, amino groups, peptide carbonyl oxygen atoms, and phosphate groups. Depending on the peptide sequence and available donor atoms, zinc ions may adopt mono-, bi-, or multidentate coordination modes, leading to distinct three-dimensional conformations and stability profiles. In many peptide–zinc complexes, Zn^2+^ serves as a molecular bridge that links distant functional groups within the peptide chain, promoting peptide folding and the formation of compact conformations. Representative computational studies have demonstrated that acidic amino acid residues such as Asp and Glu frequently coordinate zinc through carboxyl oxygen atoms, forming stable tetrahedral or bidentate structures accompanied by five- or six-membered chelate rings. For example, Liu et al. reported that zinc binding in sea cucumber-derived peptides primarily involved glutamate carboxyl groups and peptide carbonyl oxygen atoms, generating a stable tetrahedral coordination environment supported by DFT calculations [4]. Similarly, molecular modeling of rapeseed-derived zinc-chelating peptides revealed that zinc binding induced local conformational rearrangement and promoted energetically favorable bidentate coordination structures [23]. In addition to carboxyl-mediated coordination, mixed amino/carboxyl coordination has also been widely observed. Molecular dynamics simulations performed by Wang et al. demonstrated that zinc ions simultaneously interacted with *N*-terminal amino groups, glutamate carboxyl groups, and peptide carbonyl oxygen atoms, resulting in a more compact folded conformation with enhanced structural stability [17]. Such mixed coordination modes may increase resistance to gastrointestinal dissociation and improve zinc transport efficiency during intestinal absorption. Phosphopeptides exhibit a distinct coordination pattern due to the presence of phosphorylated serine residues. Multiple phosphate oxygen atoms can simultaneously interact with Zn^2+^, generating multidentate coordination networks with substantially higher binding affinity than conventional carboxyl-mediated complexes. Recent studies on β-casein phosphopeptide–zinc complexes suggested that phosphate groups may form cage-like coordination structures surrounding zinc ions, thereby enhancing zinc retention, gastrointestinal stability, and bioavailability [25].

## 5. Intestinal Transport and Zinc Bioavailability

Intestinal absorption is the central determinant of zinc bioavailability and ultimately governs the nutritional efficacy of peptide-based zinc delivery systems [53]. Unlike inorganic zinc salts, whose absorption is highly susceptible to precipitation, competitive inhibition, and luminal environmental fluctuations, peptide–zinc complexes may utilize multiple transport pathways that collectively enhance zinc retention and epithelial uptake [54]. The intestinal transport of ZCPs is not a passive diffusion process but rather a dynamic event involving transporter regulation, epithelial barrier interaction, gastrointestinal digestion, mucus penetration, and gut microenvironment modulation [55].

### 5.1. ZIP/ZnT-Mediated Zinc Transport Pathways

**Figure 3:**
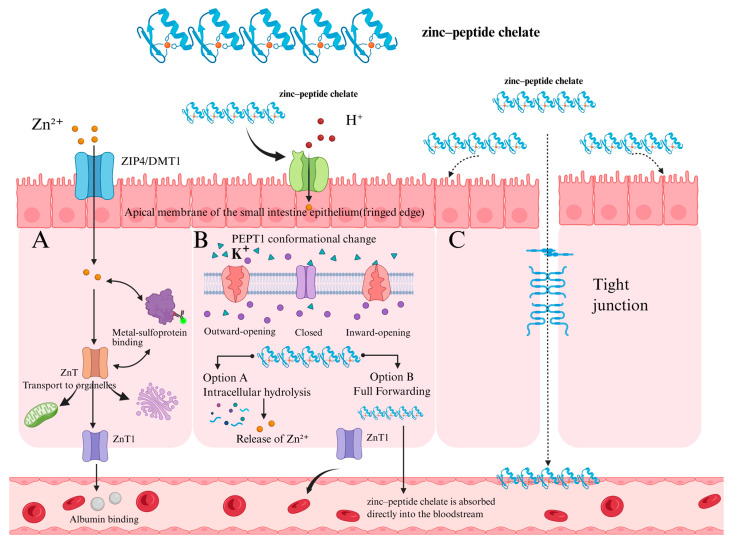
Proposed Intestinal Absorption and Transport Mechanisms of Peptide–Zinc Chelates. (**A**) ZIP/ZnT-Mediated Zinc Transport Pathways. (**B**) PepT1-Mediated Transport of Peptide–Zinc Complexes. (**C**) Paracellular Transport and Tight Junction Regulation. Solid arrows indicate the direction of transport or intracellular trafficking; curved arrows indicate intracellular redistribution or regulatory interactions; dashed arrows represent proposed or hypothetical transport pathways; the vertical dashed line denotes the paracellular route across the tight junction.

Zinc homeostasis in intestinal epithelial cells is tightly regulated by the coordinated activity of the ZIP (Zrt/Irt-like protein, SLC39) and ZnT (zinc transporter, SLC30) transporter families, which collectively control zinc uptake, intracellular trafficking, sequestration, and systemic export [56]. Among these transporters, ZIP4 is considered the principal apical transporter responsible for dietary zinc absorption in the small intestine [57]. Under physiological conditions, free Zn^2+^ released within the intestinal lumen is transported into enterocytes primarily through ZIP4 [58], whose expression is dynamically regulated according to systemic zinc status. Zinc deficiency markedly upregulates ZIP4 abundance to enhance absorption efficiency, whereas excessive zinc intake promotes transporter internalization and degradation to prevent zinc overload [59]. Most current understanding of zinc transport regulation in the context of peptide–zinc complexes is derived from cell-based and animal studies, rather than human clinical investigations. In vitro experiments using Caco-2 monolayers and ex vivo intestinal models have shown that peptide–zinc complexes may improve apparent zinc uptake by maintaining zinc solubility, reducing precipitation with dietary inhibitors, and generating localized zinc enrichment near the brush border membrane (Figure 3A) [60,61]. Similarly, animal studies suggest that peptide-based zinc delivery systems may enhance systemic zinc status compared with inorganic zinc salts under certain dietary conditions. However, it is important to emphasize that direct evidence demonstrating specific modulation of ZIP4 or other ZIP/ZnT transporters by intact peptide–zinc complexes remains limited. Most studies infer transporter involvement indirectly based on changes in zinc uptake or transporter expression levels, rather than direct molecular interaction evidence. Therefore, whether peptide–zinc complexes actively regulate ZIP/ZnT transporter function, or whether observed effects are secondary to improved zinc solubility and reduced luminal antagonism, remains unresolved. An additional limitation is that many experimental systems do not fully replicate physiological zinc absorption conditions. In particular, Caco-2 monolayers lack key components of the intestinal environment, including mucus layers, immune signaling, microbial competition, and dynamic zinc fluxes. As a result, transporter expression patterns and zinc uptake kinetics observed in vitro may not accurately reflect in vivo intestinal physiology. From a mechanistic perspective, the most plausible interpretation at present is that peptide–zinc complexes primarily influence ZIP/ZnT-mediated zinc homeostasis indirectly, by modulating zinc speciation, stability, and local concentration gradients at the intestinal surface, rather than directly altering transporter protein activity. Once zinc is released or dissociated from peptide complexes, free Zn^2+^ is then taken up predominantly via ZIP4, followed by intracellular redistribution mediated by ZnT1 and metallothioneins [62]. Furthermore, emerging evidence suggests that intracellular zinc trafficking may differ depending on the initial chemical form of zinc exposure (ionic zinc vs. peptide-bound zinc), potentially leading to differences in transient zinc buffering, cytoplasmic signaling responses, and metallothionein induction [63]. However, these observations remain preliminary and are primarily derived from experimental models.

### 5.2. PepT1-Mediated Transport of Peptide–Zinc Complexes

In addition to the classical zinc transport pathways, studies suggest that peptide–zinc complexes may interact with the intestinal oligopeptide transport system (particularly peptide transporter 1, PepT1), thereby potentially enhancing the efficiency of zinc uptake by intestinal epithelial cells (Figure 3B) [55]. Peptide Transporter 1 is a proton-coupled transporter highly expressed in the small intestine, primarily mediating the absorption of dietary dipeptides and tripeptides produced during protein digestion [64]. Currently, evidence supporting the involvement of peptide transporter 1 in peptide–zinc transport primarily comes from in vitro studies using a human colorectal adenocarcinoma Caco-2 cell monolayer model and transporter inhibition experiments. Reports indicate that the uptake of low-molecular-weight peptide–zinc complexes exhibits saturable kinetics, and that the addition of known substrates of peptide transporter 1 partially reduces its transport efficiency, suggesting that the peptide transport pathway may be involved in zinc uptake [65,66]. These findings suggest that some peptide–zinc complexes may temporarily maintain their coordination structure or exist in a dynamic coordination state during transepithelial transport, thereby potentially facilitating cellular zinc uptake. However, there is currently no direct in vivo evidence demonstrating that intact peptide–zinc complexes can cross the intestinal epithelium via the peptide transporter 1 under physiological conditions. The vast majority of existing studies employ simplified epithelial monolayer cell models, which cannot fully replicate key physiological factors such as proteolysis in the intestinal lumen, the mucus barrier, the gut microbiota, endogenous metal-binding ligands, and intestinal fluid kinetics. Therefore, it is currently unclear whether peptide–zinc complexes are absorbed as intact coordination complexes or dissociate into free zinc ions and peptide fragments prior to or during epithelial transport. Even if some complexes enter intestinal epithelial cells in their intact coordination form, it remains unclear whether these intact complexes can be directly utilized by the cells in a physiologically meaningful manner. Intracellular zinc homeostasis is strictly regulated by a dynamic buffering network composed of metallothioneins, zinc transporters, and various endogenous zinc-binding proteins. Intact peptide–zinc complexes cannot remain stable in the cytoplasm for extended periods. Furthermore, intracellular acidification, ligand exchange reactions, and competitive binding with endogenous zinc-binding molecules promote the gradual dissociation of zinc from the peptide ligand. The dissociated zinc enters the intracellular labile zinc pool and is subsequently redistributed to various zinc-dependent enzymes, transcription factors, and signaling proteins. Based on this mechanism, peptide ligands have traditionally been regarded as primarily serving as transient delivery carriers to enhance zinc solubility, stability, and epithelial transport efficiency, rather than as long-term intracellular carriers of bioactive zinc. A few studies have reported that cytoplasmic peptide–zinc chelates can maintain their intact coordination structure within cells, acting as a dynamic intracellular zinc buffer to prevent acute cytotoxicity caused by the sudden accumulation of free zinc ions. Zinc ions are slowly and stepwise released from the intact peptide–zinc complex, continuously supplying zinc to metal enzyme cofactors, precisely maintaining cellular zinc homeostasis while avoiding zinc overload stress responses [67]. Another study reported that intact peptide–zinc nanocomposites penetrate the intestinal epithelial cell membrane via oligopeptide transporters without completely dissociating in the extracellular space. Once inside the cell, the undissociated chelate can directly interact with zinc-dependent metalloproteins and intracellular antioxidant signaling pathways (Nrf2/HO-1), thereby mitigating oxidative damage and maintaining the catalytic activity of metalloproteases [63]. However, to date, no studies have directly tracked the intracellular transport process, dissociation kinetics, or metabolic fate of intact peptide–zinc complexes following intestinal absorption. Furthermore, it remains unclear whether specific peptide sequences possess sufficient structural stability to deliver zinc to specific intracellular regions, nor has it been determined whether peptide-mediated zinc delivery has biological effects beyond enhancing zinc bioavailability. Future research should rely on transporter-specific gene knockout models, organoid systems, dual-isotope tracing techniques, and rigorously designed human intervention trials to determine whether a physiological transport process for intact peptide–zinc complexes exists, and whether their intracellular metabolic fate can produce biological functions beyond enhancing zinc bioavailability.

### 5.3. Paracellular Transport and Tight Junction Regulation

In addition to transcellular transport pathways, paracellular transport is considered a potential auxiliary pathway for zinc absorption, a role that is particularly prominent when epithelial barrier integrity is compromised [68]. The paracellular pathway is primarily regulated by the tight junction complex, which includes claudins, proteins of the sealingin family, and occludin; these proteins collectively determine epithelial permeability and intestinal barrier function (Figure 3C) [69]. In vitro experiments and animal studies have shown that certain bioactive peptides can regulate the expression of tight junction proteins under conditions of inflammation or oxidative stress, thereby improving epithelial barrier integrity [70]. At the same time, zinc, as an essential trace element, plays a role in maintaining the structural stability of tight junctions and epithelial homeostasis. Based on these findings, some researchers have proposed that peptide–zinc complexes may synergistically regulate the intestinal barrier, thereby influencing the paracellular transport of zinc. Currently, the theoretical basis for this hypothesis derives primarily from cellular models (such as human colorectal adenocarcinoma cell models and inflammatory epithelial cell models) or chemically induced animal disease models, and lacks experimental support at the human physiological level. Such experimental systems typically involve artificially disrupting the barrier structure, making it difficult to accurately replicate the physiological state of normal intestinal epithelium. Therefore, the actual extent to which tight junction regulation affects zinc absorption under healthy physiological conditions remains unclear. Furthermore, while some studies have found that treatment with peptides or peptide–zinc complexes leads to increased expression levels of tight junction proteins, these findings are merely correlational, and the underlying mechanisms of action remain unclear. It is currently impossible to determine whether these changes in protein expression directly increase the permeability of paracellular transport for zinc or are secondary effects resulting from reduced inflammation and alleviated oxidative stress. At the same time, excessive regulation of tight junction permeability may compromise epithelial integrity [71]. There are currently no direct quantitative in vivo human studies to determine whether peptide–zinc complexes significantly enhance zinc absorption efficiency via the paracellular diffusion pathway. Existing detection methods cannot accurately distinguish between transmembrane and paracellular zinc transport fluxes under normal dietary physiological conditions, further complicating the elucidation of the underlying mechanisms. Paracellular transport is an auxiliary absorption mechanism dependent on environmental conditions; it plays a role in zinc absorption only under pathological conditions or when the barrier is compromised, and is not the dominant or universally applicable zinc uptake pathway in healthy individuals. Future research should combine novel intestinal models, real-time permeability tracing technologies, and human intervention trials to elucidate the actual contribution of tight junction regulation to enhancing the bioavailability of peptide–zinc complexes.

### 5.4. Gut Microenvironment and Zinc Bioavailability

The intestinal microenvironment plays a critical role in regulating the absorption, transport, and physiological utilization of peptide–zinc complexes. Beyond epithelial transporter activity alone, factors including gut microbiota composition, mucus barrier properties, luminal metabolites, and intestinal inflammatory status collectively influence zinc bioavailability and gastrointestinal homeostasis [72]. Peptide–zinc complexes may interact dynamically with the gut ecosystem in ways that differ substantially from conventional zinc salts. The gut microbiota participates extensively in mineral metabolism and nutrient competition, with certain microbial species competing with the host for luminal zinc while others indirectly enhance zinc absorption through short-chain fatty acid (SCFA) production, luminal pH regulation, and maintenance of epithelial barrier integrity. Compared with high-dose inorganic zinc supplementation, which may disrupt microbial balance and induce dysbiosis, peptide-mediated zinc delivery may provide more controlled zinc availability and reduced microbial toxicity. In addition, some food-derived peptides themselves possess prebiotic or antimicrobial properties capable of modulating microbial composition and improving intestinal homeostasis [73].

The mucus layer further represents an important but often overlooked determinant of zinc transport efficiency. Physicochemical characteristics of peptide–zinc complexes, including molecular size, charge distribution, hydrophobicity, and aggregation behavior, strongly affect mucus penetration and epithelial accessibility [74]. Moderately amphiphilic and low-molecular-weight complexes generally exhibit superior mucus diffusion capacity, whereas highly charged or aggregated structures may become trapped within the mucus network and experience reduced transport efficiency. Moreover, local mucus conditions may influence zinc dissociation kinetics and coordination stability near the epithelial surface. Intestinal inflammatory status also significantly affects zinc absorption dynamics. Chronic inflammation can disrupt epithelial integrity, alter transporter expression, increase oxidative stress, and impair nutrient utilization, thereby contributing to zinc deficiency in conditions such as inflammatory bowel disease, metabolic inflammation, aging-associated gut dysfunction, and chronic infection [75]. Importantly, many peptide–zinc complexes possess antioxidant and anti-inflammatory activities capable of suppressing inflammatory signaling pathways, stabilizing tight junctions, and restoring intestinal barrier function [76]. These multifunctional properties may improve zinc absorption indirectly by optimizing the intestinal microenvironment itself.

### 5.5. Limitations of Current Bioavailability Evaluation Models

Most existing studies rely heavily on simplified in vitro models and animal experiments that cannot fully replicate the complexity of the human gastrointestinal environment (Table 2). Caco-2 cell monolayers, one of the most widely used in vitro models for assessing zinc transport and peptide absorption, lack several critical physiological components, including mucus layers, immune cells, intestinal microbiota, dynamic luminal flow, and complex nutrient interactions [77]. Consequently, transport efficiencies observed under these conditions may substantially overestimate actual in vivo bioavailability. Similarly, static gastrointestinal digestion models are unable to reproduce the continuously changing pH conditions, enzymatic gradients, competitive ion interactions, and coordinated digestive processes occurring within the human intestine. Animal models, particularly rodent zinc-deficiency systems, also present important translational limitations. Many studies employ severe zinc-deficiency conditions or unrealistically high supplementation doses that differ markedly from the chronic marginal zinc deficiency commonly observed in humans. In addition, species-specific differences in intestinal physiology, microbiota composition, transporter regulation, metabolism, and dietary patterns complicate extrapolation of animal data to clinical nutritional settings. Another major limitation in the field is the continued overreliance on zinc-chelation rate or binding affinity as the primary indicator of peptide performance. Although zinc-binding capacity provides useful preliminary information regarding coordination potential, high chelation affinity does not necessarily correlate with improved intestinal absorption or physiological utilization [14]. Zinc bioavailability is instead determined by multiple interconnected factors, including gastrointestinal stability, digestion behavior, transporter accessibility, mucus penetration, microbiota interactions, dissociation kinetics, intracellular trafficking, and controlled zinc release. Therefore, future research should establish integrated and physiologically relevant evaluation systems combining advanced gastrointestinal simulation, epithelial transport analysis, microbiome assessment, in vivo nutritional validation, and systems-level bioavailability modeling. Such multidimensional approaches will be essential for accurately assessing the true nutritional efficacy and translational potential of peptide-based zinc delivery systems.

## 6. Biological Functions of Zinc-Binding Peptides

The physiological effects of ZCPs are not limited to their basic function as zinc delivery carriers. Relevant studies have shown that dietary peptides bound to metallic mineral elements can regulate oxidative stress [83,84,85], inflammation [86,87], intestinal homeostasis [88], metabolic balance [89], and blood sugar regulation [90], thereby exerting a variety of biological functions. This suggests that zinc–peptide chelates may also have the potential to perform multiple biological functions. Studies indicate that these biological functions of zinc-chelated peptides primarily stem from enhanced zinc bioavailability, while some effects may also arise from the biological activity of the ligand peptides themselves, acting in concert [6]. As for whether the formation of ZCPs gives rise to independent new activities, direct evidence is currently lacking. Although some studies have tentatively suggested that structural changes induced by chelation may confer unique biological properties, there is still a lack of direct experimental evidence to clearly distinguish this effect from those resulting from improved zinc delivery efficiency and the biological activity of the peptides themselves [91].

### 6.1. Regulation of Oxidative Stress and Redox Homeostasis

Oxidative stress is a major pathological factor involved in aging, metabolic disorders, chronic inflammation, intestinal dysfunction, and neurodegenerative diseases [92]. Preclinical studies have found that peptide–zinc chelates may exert antioxidant effects through a synergistic interaction between the zinc ions they coordinate and the intrinsic biological activity of the peptide fragments. Zinc itself contributes to redox homeostasis through multiple mechanisms, including stabilization of cellular membranes, inhibition of transition metal-catalyzed oxidative reactions, maintenance of protein structural integrity, and regulation of antioxidant enzymes such as superoxide dismutase (SOD) [93].

Meanwhile, many food-derived peptides possess intrinsic antioxidant activities due to the presence of amino acid residues such as histidine, tyrosine, methionine, cysteine, and hydrophobic amino acids that can directly scavenge ROS and suppress oxidative damage [94]. Studies have demonstrated that peptide–zinc complexes can moderately upregulate the activities of antioxidant enzymes including SOD, catalase (CAT), and glutathione peroxidase (GSH-Px), while alleviating MDA accumulation and reducing intracellular reactive ROS levels [95]. In many cases, these antioxidant effects are stronger than those observed with zinc salts or peptides alone, indicating functional synergy between zinc coordination and peptide-mediated bioactivity.

**Figure 4:**
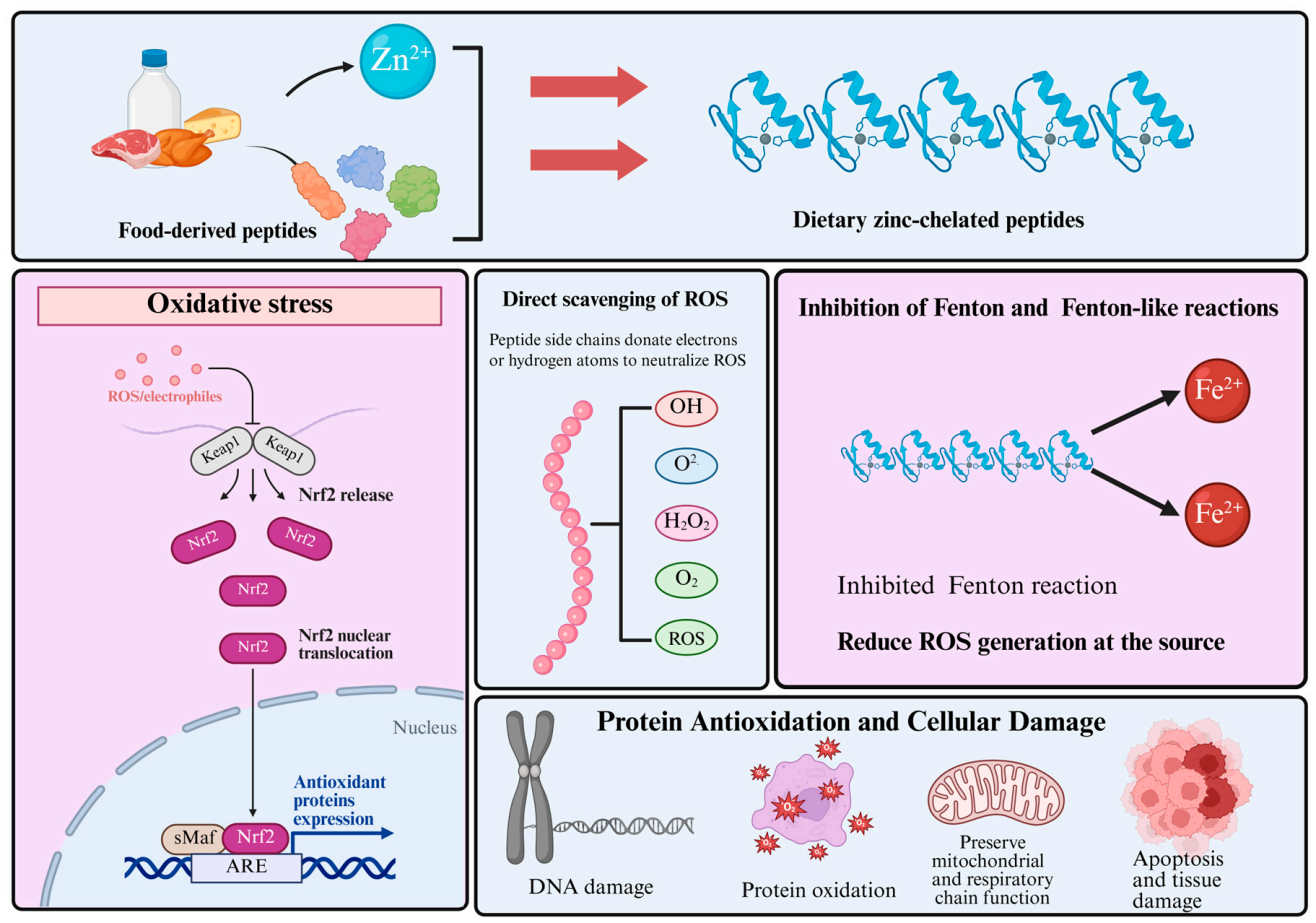
Antioxidant mechanisms and cytoprotective effects of dietary zinc-chelated peptides.

In addition to direct antioxidant effects, peptide–zinc complexes may regulate redox homeostasis through activation of the Nuclear Factor Erythroid 2-Related Factor 2 (Nrf2) signaling pathway [96], one of the central cellular defense systems against oxidative stress. Activation of Nrf2 promotes the transcription of antioxidant and cytoprotective genes, including Heme Oxygenase-1 (HO-1), NAD(P)H quinone dehydrogenase 1 (NQO1), and glutathione-related enzymes, thereby enhancing cellular resistance to oxidative injury (Figure 4). Emerging evidence suggests that peptide–zinc complexes may activate Nrf2 signaling more effectively than conventional zinc supplements due to improved intracellular zinc availability combined with peptide-mediated modulation of redox-sensitive pathways and mitochondrial function [63]. Nevertheless, there is currently no conclusive clinical evidence to confirm that these antioxidant effects translate into measurable improvements in biomarkers of oxidative stress in humans. Therefore, the observed effects should be regarded as preclinical indicators rather than established physiological outcomes. Although some studies have suggested that activation of the Nrf2 signaling pathway may be a potential mechanism, most of the supporting data consist of indirect evidence and originate from simplified experimental systems.

### 6.2. Immunomodulatory and Anti-Inflammatory Activities

**Figure 5:**
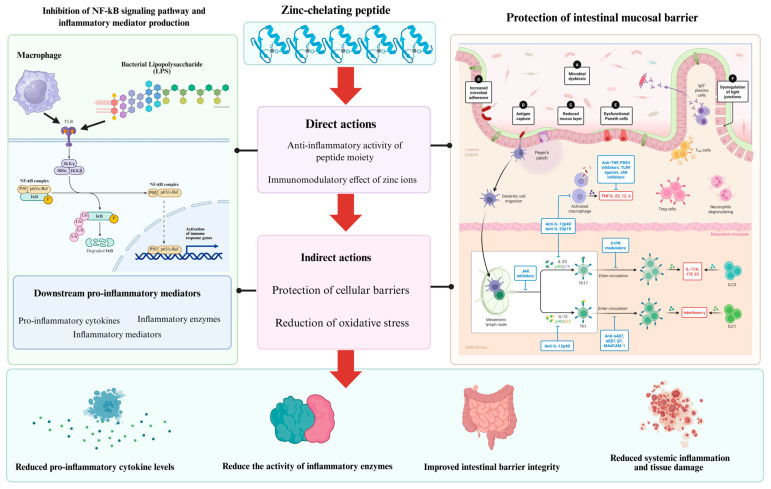
Anti-inflammatory mechanisms and intestinal barrier-protective effects of zinc-chelating peptides. Zinc-chelating peptides suppress inflammatory signaling through both direct and indirect mechanisms. Direct actions include the anti-inflammatory activity of the peptide moiety and the immunomodulatory effects of zinc ions, whereas indirect actions involve protection of cellular barriers and reduction of oxidative stress. These coordinated effects inhibit NF-κB activation, preserve intestinal mucosal integrity, reduce pro-inflammatory mediator production, and ultimately alleviate systemic inflammation and tissue damage. Symbols: red downward arrows (↓), sequential progression of biological effects; black connecting lines, mechanistic associations between different biological processes.

Zinc is essential for maintaining immune homeostasis and regulating inflammatory responses [97]. Peptide–zinc complexes may exert immunomodulatory and anti-inflammatory effects that extend beyond simple zinc supplementation [98]. Zinc deficiency is strongly associated with impaired innate and adaptive immunity, chronic inflammation, oxidative imbalance, and increased susceptibility to infection, whereas restoration of zinc homeostasis can improve immune cell function and inflammatory regulation [99]. At the same time, many food-derived peptides possess intrinsic anti-inflammatory activities, enabling peptide–zinc complexes to function as multifunctional bioactive systems through the combined actions of coordinated zinc ions and peptide moieties [100]. Peptide–zinc complexes preliminarily demonstrated anti-inflammatory activity in animal models, inhibiting the secretion of pro-inflammatory mediators, including TNF-α, IL-1β, and IL-6 [101]. Oyster peptide–zinc complexes modulate the TLR4/NF-κB pathway to reduce the levels of NF-κB, TLR4, COX-2, iNOS, TNF-α, IL-6, and IFN-γ, while increasing IL-10 levels to exert anti-inflammatory effects. They also maintain intestinal barrier function and alleviate inflammatory damage by promoting mucus secretion, repairing goblet cells, and enhancing the expression of tight junction proteins (Figure 5) [76]. In addition, the chelate formed by chito-glycosylated oyster peptides and zinc can alleviate intestinal inflammatory damage caused by zinc deficiency and improve gut dysbiosis [102]. Peptide-mediated zinc delivery may provide more stable intracellular zinc availability and reduce the risk of acute zinc overload compared with conventional inorganic zinc salts, thereby supporting more controlled immune regulation [103]. However, the vast majority of experiments were conducted under pathological conditions or in artificially induced inflammatory environments, and thus cannot reflect the normal physiological immune regulatory processes in healthy humans. Furthermore, most studies failed to distinguish whether the observed effects stemmed from zinc supplementation, the intrinsic bioactivity of the peptides, or an indirect reduction in oxidative stress levels. The key issue is that there is currently a lack of clinical trials evaluating changes in human immune markers following supplementation with peptide–zinc complexes; consequently, the value of existing research findings for clinical translation is limited.

### 6.3. Bone Formation and Muscle Protection

**Figure 6:**
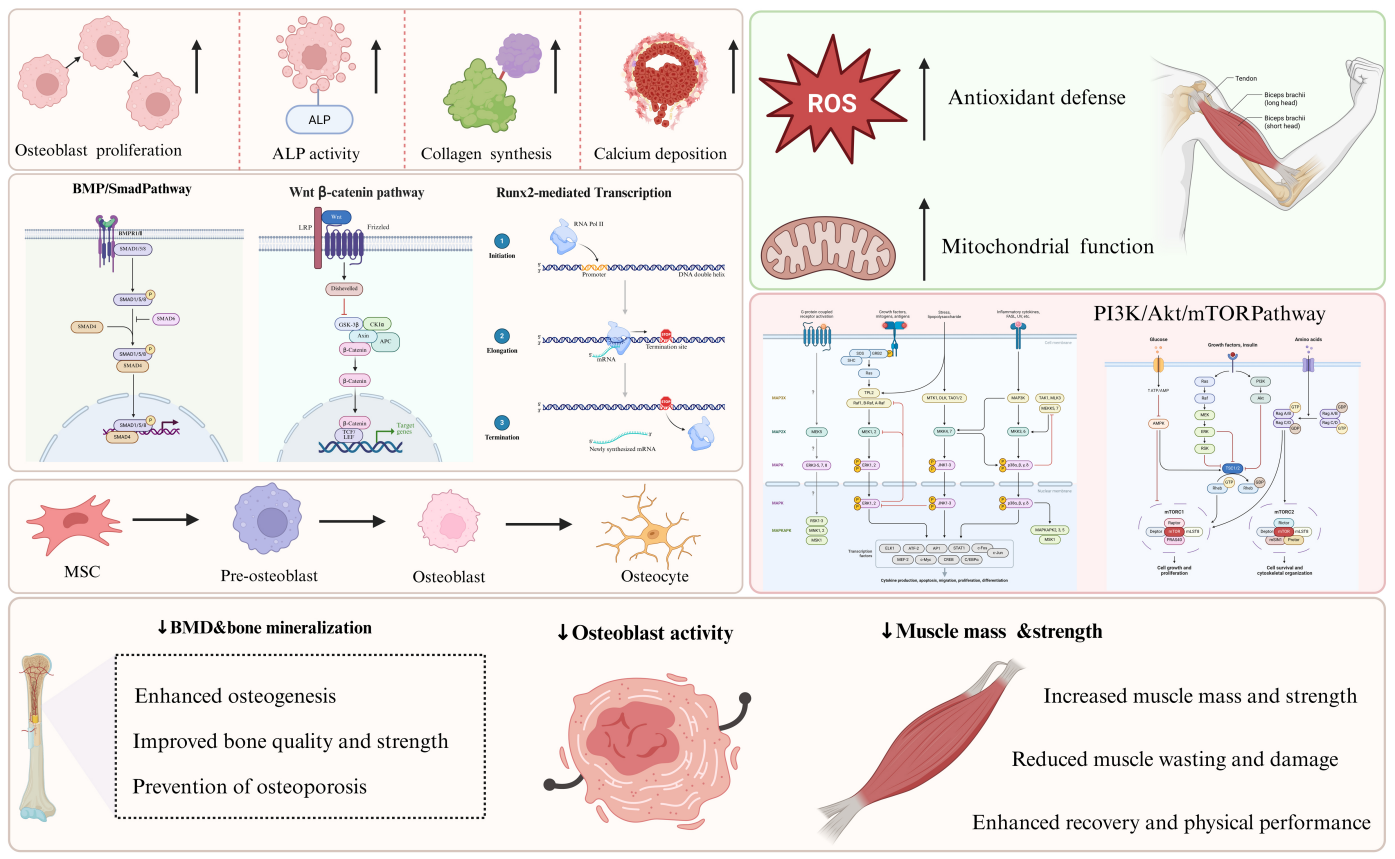
Mechanisms underlying the osteogenic and muscle-protective effects of peptide–zinc complexes. Peptide–zinc complexes promote osteogenesis by enhancing osteoblast proliferation, differentiation, extracellular matrix formation, mineralization, and osteogenic signaling while simultaneously improving antioxidant capacity, mitochondrial function, and skeletal muscle homeostasis. These coordinated effects ultimately contribute to increased bone mineral density, improved bone quality, and enhanced muscle mass and strength. Symbols: increased/activated (↑); decreased/inhibited (↓); biological progression (→), differentiation, or signaling flow.

Zinc plays a fundamental role in skeletal development, collagen synthesis, osteoblast differentiation, muscle protein metabolism, and tissue repair, and chronic zinc deficiency is closely associated with osteoporosis, impaired bone mineralization, sarcopenia, and reduced physical performance, particularly in aging populations [104]. A study on peptide chelates of essential trace metals found that, among various essential trace metals, zinc compounds exert a unique anabolic effect on bone metabolism in the femoral shaft of rats subjected to skeletal unloading; zinc-chelated dipeptides may stimulate bone protein synthesis through a protein kinase mechanism [105]. The ability of peptide–zinc complexes to promote osteoblast proliferation, increase alkaline phosphatase activity, and stimulate collagen synthesis may be related to osteogenic signaling pathways (Figure 6). In addition to improving zinc delivery efficiency, certain food-derived peptides themselves possess intrinsic osteogenic bioactivity, further contributing to bone formation and mineral metabolism [106]. Marine-derived peptide–zinc complexes have attracted particular attention due to their combined mineral-binding capacity and potential bone-protective effects. Beyond skeletal regulation, supplementing with zinc amino acids can significantly reduce the incidence of muscle disorders in broiler chickens, increase the dry matter and protein content of their meat, and maintain muscle health [107]. Aging-associated zinc deficiency contributes to muscle wasting, oxidative damage, and impaired recovery capacity [108]; peptide-mediated zinc delivery may have potential value in maintaining intracellular zinc homeostasis in muscle tissue and alleviating oxidative stress and inflammatory damage in muscle tissue. However, nearly all of the above findings are derived from in vitro osteoblast models and animal studies; there are currently no clinical trials confirming that supplementation with peptide–zinc complexes can improve bone density, reduce the risk of fractures, or maintain muscle mass in humans. Furthermore, most of the signaling pathways frequently cited in existing studies are derived solely from preclinical models, and their actual role in nutritional interventions in humans remains to be verified.

### 6.4. Metabolic Regulation and Glucose Homeostasis

Zinc contributes to pancreatic β-cell function by stabilizing insulin crystal formation and regulating insulin receptor signaling pathways, while zinc deficiency impairs glucose metabolism and exacerbates oxidative stress and chronic inflammation associated with metabolic dysfunction [109]. Food-derived bioactive peptides modulate lipoprotein signaling, inhibit α-glucosidase activity, and improve insulin sensitivity, which may contribute to glycemic regulation [110,111]. In addition to glucose metabolism, studies have shown that dietary fiber–zinc complexes may also influence lipid metabolism and obesity-related metabolic inflammation [112]. Following supplementation with zinc peptides, lipid peroxidation levels decrease, the antioxidant status of the liver improves, and signaling pathways related to lipid metabolism are modulated [113]. The underlying mechanisms may include activation of AMPK, inhibition of pro-inflammatory cytokine production, enhancement of mitochondrial energy metabolism, and mitigation of metabolic damage caused by oxidative stress. Compared with conventional inorganic zinc supplements, peptide-mediated zinc delivery may provide more stable zinc homeostasis and reduced gastrointestinal irritation, potentially improving long-term metabolic regulation [114]. Nevertheless, most of the aforementioned studies employed acute or short-term intervention models, which cannot simulate the actual state of chronic metabolic diseases in the human body. In addition, most of the evidence suggesting that the peptide–zinc complex can directly regulate key metabolic pathways—such as AMPK activation and insulin receptor signaling—is indirect, and there are significant discrepancies in the experimental results across different studies. To date, there are no rigorously designed human intervention trials confirming that this complex possesses hypoglycemic effects or long-term metabolic protective effects; existing conclusions are limited to observations from preclinical experiments.

### 6.5. Neuroprotective and Cognitive Regulatory Potential

Zinc is highly enriched in the central nervous system and plays critical roles in synaptic transmission, neuronal signaling, and cognitive function [115]. Both zinc deficiency and zinc overload can disrupt neuronal homeostasis and contribute to neurodegenerative disorders [116]. Metal-binding peptides exert neuroprotective effects by regulating metal ion homeostasis in the brain and alleviating amyloid-related neuronal damage [117]. In a zinc-deficient neuronal damage model, this glycosylated peptide–zinc complex alleviates neuronal oxidative stress and reduces zinc-deficiency-induced cellular damage; this effect is partly attributed to enhanced zinc bioavailability and the intrinsic antioxidant capacity of the glycosylated oyster peptide fragment itself [118].

## 7. Emerging Technologies and Future Perspectives

Although food-derived ZCPs exhibit promising zinc delivery efficiency and multifunctional bioactivities, several limitations still hinder their clinical and industrial translation. Current studies remain largely dependent on empirical peptide screening, simplified in vitro models, and indirect mechanistic evidence, while systematic evaluation of intestinal transport, controlled zinc release, and long-term physiological effects is still insufficient. Future research should therefore focus on mechanism-driven design, physiologically relevant bioavailability evaluation, and precision nutritional applications.

### 7.1. AI-Assisted Peptide Design and Computational Prediction

**Figure 7:**
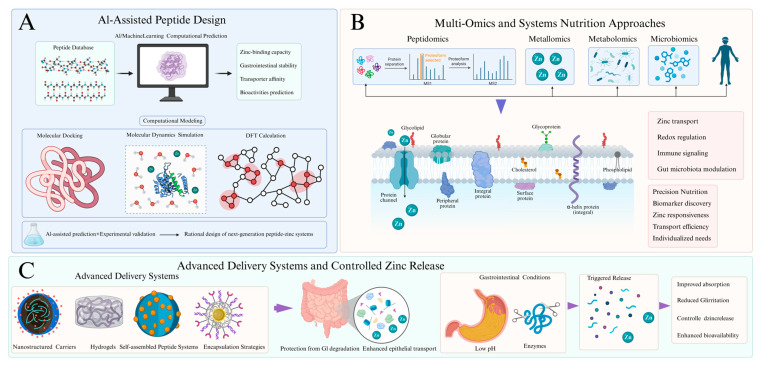
Emerging technologies and future development framework of food-derived zinc-chelating peptides. (**A**) AI-assisted peptide design and computational prediction for optimized zinc-binding and transport efficiency. (**B**) Multi-omics and systems nutrition approaches for mechanism elucidation and personalized zinc nutrition. (**C**) Advanced delivery systems enabling controlled zinc release and enhanced gastrointestinal stability.

Artificial intelligence (AI) and computational modeling are emerging as powerful tools for accelerating the discovery and optimization of ZCPs. Machine learning algorithms can predict zinc-binding capacity, gastrointestinal stability, transporter affinity, and multifunctional bioactivities based on peptide sequence characteristics, thereby reducing experimental workload and improving screening efficiency. In parallel, molecular docking, molecular dynamics simulation, and density functional theory (DFT) provide important insights into coordination sites, binding stability, and zinc release behavior at the molecular level (Figure 7A). Integration of AI-assisted prediction with experimental validation may facilitate the rational development of next-generation peptide–zinc systems with optimized transport efficiency and biological functionality.

### 7.2. Multi-Omics and Systems Nutrition Approaches

The physiological effects of peptide–zinc complexes involve complex interactions among zinc transport, redox regulation, immune signaling, and gut microbiota modulation. Multi-omics technologies, including peptidomics, metallomics, metabolomics, and microbiomics, provide new opportunities for elucidating structure–function relationships and systemic nutritional effects. In particular, integrated systems nutrition approaches may help identify biomarkers associated with zinc responsiveness, intestinal transport efficiency, and individualized nutritional requirements (Figure 7B). However, standardization of omics integration and physiologically relevant validation models remains a major challenge for future research.

### 7.3. Advanced Delivery Systems and Controlled Zinc Release

Advanced delivery technologies may further improve the stability, bioaccessibility, and targeting efficiency of peptide–zinc complexes. Nanostructured carriers, self-assembled peptide systems, hydrogels, and encapsulation strategies have shown potential for protecting peptide–zinc complexes against gastrointestinal degradation and enhancing epithelial transport. In addition, stimuli-responsive delivery systems capable of pH- or enzyme-triggered zinc release may improve intestinal absorption efficiency while minimizing gastrointestinal irritation and uncontrolled zinc dissociation (Figure 7C). Future development of smart zinc delivery systems may enable multifunctional nutritional regulation and targeted health intervention.

## 8. Conclusions

Food-derived ZCPs have emerged as highly promising multifunctional nutritional systems that combine efficient zinc delivery with diverse biological activities. Through the coordinated regulation of zinc solubility, gastrointestinal stability, transporter accessibility, intestinal barrier integrity, and interactions with the gut microenvironment, peptide-mediated zinc delivery systems may overcome several limitations associated with conventional zinc supplements. Beyond enhancing zinc bioavailability, peptide–zinc complexes exhibit antioxidant, anti-inflammatory, immunomodulatory, metabolic regulatory, and gut-protective activities through the synergistic effects of coordinated zinc ions and bioactive peptide structures. Future studies integrating advanced gastrointestinal models, multi-omics technologies, and AI-assisted peptide design will be essential for elucidating structure–function relationships and optimizing translational applications.

## Figures and Tables

**Table 1:** Sequences of high-value zinc-chelating peptides derived from food sources. note: Ala, Alanine; Arg, Arginine; Asp, aspartic acid; Asn, Asparagine; Gly, Glycine; Gln, Glutamine; Glu, Glutamic acid; His, Histidine; Ile, Isoleucine; Leu, Leucine; Trp, Tryptophan; Tyr, tyrosine; Pro, Proline; Ser, Tryptophan; Val, Valine.

Source	Enzyme	Amino Acid Sequence	Reference
Soy protein isolate	Lys-Tyr-Lys-Arg-Gln-Arg-Trp	Alkaline protease; Flavor protease	[21]
Millet bran	Ser-Asp-Asp-Val-Leu	Neutral protease	[22]
Rapeseed protein	Ala-Ser-His	Papain	[23]
Naked Oat Bran Albumin	Gly-Tyr-His-Gly-His	Trypsin; Pepsin	[24]
β-casein	Tyr-Pro-Val-Glu-Pro-Phe	trypsin	[25]
Bitter almond protein	Leu-Asp-Arg-Leu-Glu	Trypsin; Pepsin	[26]
Sea cucumber protein	Trp-Leu-Thr-Pro-Thr-Tyr-Pro-Glu	Alkaline protease	[27]
Chickpea protein	His-Lys-Glu-Arg-Val-Gln-Leu-His-Ile-Ile-Pro-Thr-Ala-Val-Gly-Lys	Pepsin	[28]
Pumpkin Seed Protein	Arg-Pro-Lys-His-Pro-Leu-Lys	Papain	[29]
Soy protein	Glu-Glu-Gly-Gln-Gln-Gln-Gly-Glu-Gln-Arg	trypsin	[19]
Coconut globulin	Phe-Asp-Glu-Glu	Flavourzyme; Alcalase	[30]
Oyster protein	Glu-His-Ala-Pro-Asn-His-Asp-Asn-Pro-Gly-Asp-Leu	/	[31]
Wheat protein	Asp-Lys-Val-Ile-Val-Pro	Papain	[32]
Oil palm kernel globulin antihypertensive peptides	Glu-Val-Pro-Gln-Ala-Tyr-Ile-Pro	Papain	[33]

**Table 2:** A useful experimental model for studying the bioavailability of zinc-chelated peptides.

Model Type	Model	Peptide Sources	Measurement	Reference
In vivo	Rat model (Zn-deficient)	Fish collagen peptides	Serum Zn, liver Zn, ALP	[78]
In vivo	Rat model	Oyster peptides	Tissue Zn, antioxidant enzymes	[16]
In vivo	Mouse model	Pumpkin seed peptides	Growth performance, Zn absorption	[79]
In vivo	Rat model	Coconut protein peptides	erum Zn, SOD, GSH-Px	[30]
In vivo	Broiler chicken model	Soy protein peptides	Growth rate, bone Zn content	[19]
In vivo	Rat model	Casein phosphopeptides	Zn retention, absorption rate	[40]
In vitro	Caco-2 monolayer model	Tilapia Collagen Peptides	Transmembrane transport (Papp), cellular uptake, and analysis of Zn transport pathways	[80]
In vitro	Caco-2 model	Oyster protein hydrolysate	Zn absorption rate, trans-epithelial transport capacity, intracellular Zn content	[81]
In vitro	Caco-2/Transwell model	Silkworm pupa protein peptides	Cell Uptake Rate, Inflammation Regulation, and Uptake Mechanisms	[82]
In vitro	Caco-2 monolayer model	Antarctic krill peptide	Digestive stability, Zn release, and transport capacity	[18]
In vitro	Caco-2 model	Casein peptides	Antioxidant activity and zinc absorption	[52]

## Data Availability

No new data were created or analyzed in this study. Data sharing is not applicable to this article.

## Abbreviations

ALP	Alkaline Phosphatase
AMPK	Adenosine Monophosphate-Activated Protein Kinase
BMP	Bone Morphogenetic Protein
CPPs	Casein Phosphopeptides
CAT	Catalase
DMT1	Divalent Metal Transporter 1
DSS	Dextran sulfate sodium
FTIR	Fourier-Transform Infrared Spectroscopy
GSH-Px	Glutathione Peroxidase
HO-1	Heme Oxygenase-1
IKK	IκB Kinase
LPS	Lipopolysaccharide
MDA	Malondialdehyde
MAPK	Mitogen-activated protein kinase
NF-κB	Nuclear Factor-κB
Nrf2	Nuclear Factor Erythroid 2-Related Factor 2
NQO1	NAD(P)H quinone dehydrogenase 1
NO	Nitric oxide
PepT1	Peptide Transporter 1
PI3K	Phosphoinositide 3-Kinase
ROS	Reactive Oxygen Species
Runx2	Runt-Related Transcription Factor 2
SCFA	Short-Chain Fatty Acid
SOD	Superoxide Dismutase
TNF-α	Tumor Necrosis Factor-α
XPS	X-ray photoelectron spectroscopy
ZCPs	Zinc-Chelating Peptides
ZIP	Zrt/Irt-Like Protein
ZnT	Zinc Transporter
